# Assessing the Effectiveness of a Mental Health Literacy Programme for Refugee Teachers in Malaysia

**DOI:** 10.21315/mjms2019.26.6.12

**Published:** 2019-12-30

**Authors:** Kok Wai Tay, Anna Wen Huey Ong, Kai Shuen Pheh, Sew Kim Low, Chee Seng Tan, Poi Kee Low

**Affiliations:** 1 Department of Psychology and Counselling, Universiti Tunku Abdul Rahman (Perak Campus), Malaysia; 2 Center for American Education, INTI International University & Colleges, Malaysia; 3 PSYCHLIVING Associates, Singapore

**Keywords:** mental health literacy, refugee, trauma, Malaysia, United Nations High Commissioner for Refugees

## Abstract

**Background:**

Children and young refugees often experience negative events that affect their mental health. Their caregivers may also be in the same predicament, implying that the teachers in schools are a potential source of help and support. However, most teachers have little understanding of mental health and are, thus, clueless in helping their students. To address this need, a newly developed one-day mental health literacy programme was conducted among 68 refugee teachers in Malaysia.

**Methods:**

Participants learned the symptoms of mental health issues among children and adolescents in the context of post-trauma, provision of early intervention, and channel for professional supports. They also answered a packet of measurements of mental health literacy before and after the programme.

**Results:**

The paired sample *t*-test showed that participants reported higher willingness to contact with people having mental health problems (*t* = 2.787, *P* = 0.008, Cohen’s *d* = 0.394), less stereotypes toward mental illness (t = 4.603, P < 0.001, d = 0.651) and a better understanding of self-help strategies (t = 2.16, P = .036, d = 0.322) than baseline.

**Conclusion:**

The results of this study offered preliminary empirical evidence on the effectiveness of the programme as a promising channel for alleviating mental health issues among refugees.

## Introduction

The Malaysian National Health and Morbidity Survey (1) reported that emotional disorder among youth had increased from 20.3% in 2006 to 29.2% in 2015 and identified adolescents (aged 15 to 19 years) and emerging adults (aged 20 to 25 years) as high-risk groups for suicide. With 175,760 registered refugees and asylum-seekers in Malaysia (45,470 of them are children aged below 18 years) (2), it is reasonable to assume refugee children and adolescents leaving their homeland to a foreign land as asylum seekers are prone to mental health issues. Indeed, studies have shown that many refugees faced post-traumatic stress, depression, and somatic complaints (3, 4) and were characterised by increased anxiety and disorderly conduct (5). Similarly, refugees aged 14 to 27 years in Malaysia reported extreme levels of anxiety, stress and depression (6). Moreover, these refugees encountered barriers to access healthcare services provided by non-governmental organisations such as the A Call to Service (ACTS), Health Equity Initiative (HEI), Kuala Lumpur Buddhist Tzu Chi free clinic and Malaysian Social Research Institute (MSRI) due to financial and language difficulties as well as their social-cultural beliefs (7).

Given the high-intensity exposure to trauma and the high prevalence rate of mental health issues among refugees, trauma-informed mental health literacy programmes among educators working with refugee children and adolescents are essentially necessary to prevent the anticipated mental health problems. Attention is particularly given to educators as some refugee children and adolescents speaking only their mother tongue will experience difficulty in communicating with clinicians. Thus, teachers who can speak both refugees’ mother tongue and local languages can help to overcome the language barrier. Indeed, refugee families are more open to child mental health services aligned with their home culture alongside teachers’ involvement (8). Besides, refugee children and adolescents (of busy working parents) who spend more time with teachers will experience psychological closeness that leads them to feel safe sharing their problems with the teachers. Therefore, teachers can help to identify at-risk refugee children and adolescents. In fact, some participants of the present study told the authors that they have found some students who required professional help but lack proper guidance to solution. Collectively, the present study was undertaken to examine effectiveness of a newly developed mental health literacy programme for refugee teachers in Malaysia.

### Mental Health Literacy Programme for Teachers

An eight-hour trauma-informed mental health literacy programme was developed by the researchers and clinical psychologists (PKS and TKW) for refugee teachers. It covers the signs and symptoms of common children’s and adolescents’ mental health issues in the context of post-trauma, provision of early intervention as well as linking concerned individuals to professional support. The programme also covers acute and chronic traumatic responses, stress, anxiety disorders and depressive disorders among children and adolescents. Early interventions of the programme would include active listening, progressive muscle relaxation, mindful breathing, mindful eating, and contingency management of behavioural issues in children and adolescents. Throughout the programme, participants learn about psychological and neurobiological effects of trauma, risks and protective factors of mental health problems, evidence-based treatment approaches as well as ways for refugees within Malaysia to get professional help. Precisely, the devised five-step action plan for supporting individuals with mental health concerns, i.e., CARE^2^ comprises: (i) C – care and concerns, (ii) A – attentive listening, (iii) R – risk review and management, (iv) E – empowerment with information, and finally (v) E – ensure professional help. The plan would incorporate a problem-based learning approach in delivery, consistent with the best practice of medical and health sciences pedagogy (9–11).

## Methods

### Participants and Procedure

The United Nations High Commissioner for Refugees (UNHCR) representatives in Malaysia took charge of recruiting potential participants from different refugee learning centres. There are over 177,000 refugees and asylum-seekers registered with UNHCR in Malaysia and 63.06% of them reside in Kuala Lumpur, Selangor, and Penang (2). Three sessions were conducted in Kuala Lumpur and Penang. These two states were selected as sites for this investigation as they both offer dense concentrations of refugee population exposed to similarly urbanised environments.

A total of 68 teachers (*M*_age_ = 30.26, SD = 10.61, 63.2% females and three participants did not reveal gender) with 1 to 9 years (*M* = 3.11, SD = 2.07) of working experience at a refugee centre participated in the programme. They are generally more educated individuals among their respective communities and many are fluent in English language. Most teachers lack pre-service as well as in-service teacher training. Meanwhile, the UNHCR and NGOs could provide structured education for 20% of refugee children and young people under the age of 18 years. The remaining 80% of these children attend community-based schools or learning centres (12).

The present study employed single group pre- and post-test design. Participants answered a packet of survey before and right after the programme. The study was approved by the Institutional Review board, and consent was obtained from all participants.

### Measurements

The following measures were presented in English. Participants indicated the extent to which they (dis)agreed with each item on a 5-point Likert scale ranging from 1 (strongly disagree) to 5 (strongly agree).

#### Mental Health Knowledge Schedule (13)

The first six questions in the Mental Health Knowledge Schedule (MHKS) assessed an individual’s stigma-related mental health knowledge (i.e., MAKS) and the remaining six questions examined classification of various conditions such as mental illnesses (i.e., recognition). Items 6, 8 and 12 were reverse scored prior to computing the overall score and subscale scores. A higher score indicates greater mental health knowledge.

#### Reported and Intended Behaviour Scale (14)

The 8-item of Reported and Intended Behaviour Scale (RIBS) assessed people’s behavioural reactions and intentions toward those with mental health problems. Participants responded to the first four dichotomous items (0: No/Don’t know versus 1: Yes) to report their past and current contacts (e.g., working with, or have worked with, someone with a mental health problem). Another four items were for them to indicate their willingness in contacting people having mental health problems in the future (e.g., willing to work with someone with a mental health problem in the future).

#### Attitudes and Knowledge about Mental Health Conditions (15)

Participants answered 12 items to indicate their attitudes and knowledge about mental health. A composite score was generated by summing the scores on each item after reverse-scoring five items. A high score indicates a more positive attitude and/or accurate knowledge.

#### Mental Health Literacy Questionnaire for Young Adult Form (16)

Participants self-reported their mental health literacy on 29 items in four dimensions, namely knowledge of mental health problems (11 items), erroneous beliefs/stereotypes (8 items), help-seeking and first aid skills (6 items), and self-help strategies (4 items). As the self-help strategies subscale had a Cronbach coefficient of 0.60, the researchers created another four items to assess the role of religiosity/spirituality, confiding to a trusted person, voluntary works, and writing down thoughts/feelings in promoting mental health in order to boost the internal consistency. Six items of the erroneous beliefs subscale required reverse-scoring prior to the computation of the total score. A high global/ subscale score indicates a better knowledge and more positive beliefs about mental disorders.

## Results

Correlations and Cronbach alpha coefficient are presented in Table 1. In pre-test, recognition was found to have a positive relationship with attitude, Mental Health Literacy Questionnaire for Young Adult Form (MHLq-YA) global score, knowledge and self-help subscales (all Pearson correlation coefficients [*r*s] > 0.29, *P*-values [*p*s] < 0.05). Similar pattern was observed for total MAKS (*r*s > 0.24, *p*s < 0.05). Intended behaviour was positively associated with attitude, knowledge and stereotype subscales of MHLq-YA (*r*s > 0.24, *p*s < 0.05). Attitude was also found positively associated with MHLq-YA global score and all subscale scores (*r*s > 0.40, *p*s < 0.01) except help-seeking subscale. In post-test, all the relationships were statistically significant (*r*s > 0.26, *p*s < 0.05) except for the relationship between stereotype and help-seeking subscales. Moreover, inspection of the alpha coefficients found that MAKS total score and its two subscales, as well as the reported behaviour subscale of RIBS and Attitudes and Knowledge about Mental Health Conditions, showed poor to unsatisfactory internal consistency in both pre- and post-measurement. Hence, the results shall be interpreted with caution.

Furthermore, several paired sample *t*-tests were conducted to examine the differences between the pre- and post-measured variables (see Table 2). Participants reported a (statistically significant) higher value than baseline in reported behaviour and intended behaviour subscales of RIBS (*t* = 2.44, *P* = 0.018, Cohen’s *d* = 0.346 for reported behaviour; *t* = 2.787, *P* = 0.008, *d* = 0.394 for intended behaviour), attitude toward mental health (*t* = 4.73, *P* < 0.001, *d* = 0.676), global scores of MHLq-YA (*t* = 3.538, *P* = 0.001, *d* = 0.50 for the original 29-item version; *t* = 3.496, *P* = 0.001, *d* = 0.494 for the 33-item version), stereotypes subscale (*t* = 4.603, *P* < 0.001, *d* = 0.651), and self-help subscale (*t* = 2.160, *P* = 0.036, *d* = 0.322 for the original 4-item version; *t* = 3.036, *P* = 0.004, *d* = 0.453 for the 8-item version). No significant difference was observed in other scales. Wilcoxon Signed Ranks Tests were also conducted to get rid of the impact of violating normality assumption. The results were found to be consistent with the paired sample *t*-tests. These results are not presented here for the sake of clarity but are available upon request to the corresponding author.

## Discussion

Refugee children and adolescents are vulnerable to mental health challenges. A mental health literacy programme was, thus, developed to teach fellow refugee teachers on signs and symptoms of common mental health issues, provision of early intervention, and procedure for seeking professional support. Results revealed that the programme has a medium effect in reducing stereotypes toward mental health as well as a small effect on willingness to approach people with mental health issues and self-help strategies. The programme is indeed helpful in instilling a positive understanding of mental health issues and ways to promote mental health. To our best knowledge, our study is the first to develop an evidence-based mental health programme for refugee teachers in Malaysia.

While the programme seems promising in advancing mental health knowledge, more studies are undoubtedly needed to improve the contents to further enhance effectiveness of the programme. For example, highlighting more of typical symptoms of mental health illness and emphasising approachable professional help could be useful in improving knowledge of mental health problems and help-seeking behaviours that did not show improvement in the present study. It will be interesting to examine if the programme is useful in helping refugee students to cope with mental health challenges. Specifically, future researchers shall follow up with participants of the programme to determine whether they could identify at-risk students and refer those students to professional supports.

## Conclusion

Finally, it is necessary to replicate the present study with larger sample size, measurements with good reliability and different populations (e.g., ordinary school teachers) to confirm its effectiveness and generalisability.

## Figures and Tables

**Table 1:** Correlation and reliability of the measurements

	1	2	3	4	5	6	7	8	9	10	11	12	13
1. MAKS	0.05	0.37**	0.82+	0.44**	0.53+	0.52+	0.59+	0.52+	0.62+	0.37**	0.51+	0.50+	0.48+
2. Recognition	0.06	0.56+	0.80+	0.27**	0.32*	0.55+	0.46**	0.46**	0.45**	0.34*	0.32*	0.31*	0.35*
3. Total MAKS	0.65+	0.80+	0.29*	0.42**	0.58+	0.52+	0.55+	0.53+	0.59+	0.47+	0.40**	0.40**	0.41**
4. Reported Bhvr	−0.20	0.23	0.05	0.49+	0.35**	0.33*	0.44**	0.44**	0.44**	0.34*	0.30*	0.45**	0.46**
5. Intended Bhvr	0.08	0.03	0.07	0.19	0.49+	0.65+	0.49+	0.46**	0.47+	0.54+	0.31*	0.60+	0.55+
6. Attitudes	0.13	0.41**	0.39**	0.20	0.35**	0.58+	0.52+	0.49+	0.53+	0.45**	0.36*	0.40**	0.39**
7. MHLq-YA (29-item)	−0.06	0.38**	0.25*	0.18	0.17	0.53+	0.87+	0.99+	0.80+	0.78+	0.72+	0.84+	0.83+
8. MHLq-YA (33-item)	−0.04	0.39**	0.27*	0.17	0.15	0.54+	1.0+	0.86+	0.79+	0.77+	0.71+	0.86+	0.87+
9. Knowledge	−0.05	0.44+	0.31*	0.23	0.25*	0.45+	0.89+	0.88+	0.72+	0.53+	0.61+	0.73+	0.74+
10. Stereotypes	−0.10	0.24	0.12	0.15	0.31*	0.52+	0.88+	0.87+	0.73+	0.84+	0.18	0.54+	0.52+
11. Help seeking	−0.11	0.18	0.07	0.02	−0.07	0.16	0.75+	0.72+	0.53+	0.39+	0.69+	0.55+	0.53+
12. Self-help (4-item)	0.08	0.30*	0.28*	0.16	0.09	0.43**	0.85+	0.87+	0.70+	0.53+	0.64+	0.80+	0.96+
13. Self-help (8-item)	0.07	0.36**	0.33*	0.15	0.07	0.41**	0.85+	0.90+	0.77+	0.54+	0.57+	0.95**	0.78+
Pre-α	0.270	0.209	0.120	0.585	0.856	0.463	0.917	0.936	0.870	0.744	0.807	0.881	0.942
Post-α	0.053	−0.323	0.204	0.587	0.912	0.699	0.907	0.919	0.899	0.701	0.883	0.874	0.927

Notes: Orthorgonal shows the correlation between the pre- and post-measured scores. Below orthorgonal shows the relationships for pre-test. Above orthorgonal shows the relationships for post-test. Pre-α: Crobanch alpha coefficient for pre-test; Post-α: Crobanch alpha coefficient for post-test.

MAKS = stigma-related mental health knowledge, Recognition = recognition of mental illnesses, Total MAKS = total score of the Mental Health Knowledge Schedule, Reported Bhvr = RIBS_Reported Behaviour subscale, Intended Bhvr = RIBS_Intended Behaviour subscale, Attitudes = attitudes and knowledges about Mental Health Conditions, MHLq-YA = Mental Health Literacy Questionnaire for Young Adult Form, Knowledge = knowledge of mental health conditions subscale, Stereotypes = erroneous beliefs/stereotypes subscale, Help seeking = first aid skills and help seeking behaviour subscale, Self-help (4-item) = the original self-help strategies subscale with 4 items, Self-help (8-item) = the original self-help strategies subscale with additional 4 new items.

* *P* < 0.05,

** *P* < 0.01,

+ *P* < 0.001

**Table 2:** Mean and standard deviation for pre- and post-measured variables, paired-sample *t*-test results, and effect size

No	Variable	*N*	Pre-		Post-	
	
			M	SD	M	SD
1	MAKS	50	22.460	2.484	22.560	2.908
2	Recognition	49	19.898	2.995	20.449	2.372
3	Total MAKS	50	42.320	3.878	42.600	5.693
4	Reported Bhvr*	50	1.220	1.314	1.700	1.418
5	Intended Bhvr*	50	15.960	4.535	17.960	5.432
6	Attitudes*	49	38.735	6.089	42.735	6.800
7	MHLq-YA (29-item)*	50	105.380	26.441	111.980	20.718
8	MHLq-YA (33-item)*	50	120.100	31.323	128.180	24.217
9	Knowledge	50	41.280	8.359	42.760	7.275
10	Stereotypes*	50	28.740	7.256	31.360	6.574
11	Help seeking	45	22.333	4.724	22.933	4.942
12	Self-help (4-item)*	45	16.244	3.909	17.000	3.097
13	Self-help (8-item)*	45	32.267	6.652	34.156	5.231

Note. MAKS = stigma-related mental health knowledge, Recognition = recognition of mental illnesses, Total MAKS = total score of the Mental Health Knowledge Schedule, Reported Bhvr = RIBS_Reported Behaviour subscale, Intended Bhvr = RIBS_Intended Behaviour subscale, Attitudes = attitudes and knowledges about mental health conditions, MHLq-YA = Mental Health Literacy Questionnaire for Young Adult Form, Knowledge = knowledge of mental health conditions subscale, Stereotypes = erroneous beliefs/stereotypes subscale, Help seeking = first aid skills and help seeking behaviour subscale, self-help (4-item) = the original self-help strategies subscale with 4 items, Self-help (8-item) = the original self-help strategies subscale with additional 4 new items.

* The difference between the pre- and post-measured mean scores was significant at 0.05 level
